# Physiotherapy Interventions for Migraine: A Systematic Review of Clinical Trials

**DOI:** 10.3390/clinpract16100192

**Published:** 2026-10-13

**Authors:** Helena Małgorzata Strachanowska, Martyna Anna Kaczmarska, Marlena Robakowska, Michał Brancewicz

**Affiliations:** 1 Faculty of Health Sciences with the Institute of Maritime and Tropical Medicine, Medical University of Gdańsk, 80-210 Gdańsk, Poland; strachelena@gumed.edu.pl; 2 Department of Public Health & Social Medicine, Faculty of Health Sciences with the Institute of Maritime and Tropical Medicine, Medical University of Gdańsk, 80-210 Gdańsk, Poland; mrobakowska@gumed.edu.pl (M.R.); m.brancewicz@gumed.edu.pl (M.B.)

**Keywords:** migraine, physiotherapy, manual therapy, exercise therapy, rehabilitation, systematic review, pain management

## Abstract

**Background**: Migraine is one of the leading causes of disability worldwide and significantly reduces quality of life. Standard pharmacological treatment is often associated with adverse effects and does not always provide satisfactory long-term outcomes. Consequently, increasing attention has been directed toward non-pharmacological interventions such as physiotherapy. The aim of this systematic review was to evaluate the effectiveness of physiotherapy used as a standalone or complementary treatment for migraine. **Methods**: A systematic review of randomized controlled trials (RCTs) published between 2015 and 2025 was conducted in accordance with PRISMA 2020 guidelines. Electronic databases (PubMed, Cochrane Library, Scopus and PEDro) were searched using predefined search strategies. Studies involving adults diagnosed with migraine who received physiotherapy interventions (e.g., manual therapy, exercise therapy) were included. Two reviewers independently screened records and extracted data. Methodological quality of included studies was assessed using the PEDro scale. **Results**: Thirteen clinical trials (including 12 RCTs) involving 1021 participants were included. The most frequently investigated interventions were manual therapy and exercise therapy. Improvements in at least one primary clinical outcome (migraine frequency, pain intensity, or disability scores such as MIDAS or HIT-6) were observed in 8 out of 13 studies. A reduction in migraine frequency was reported in 4 studies. However, results were inconsistent across trials, and several studies found no statistically significant differences between the intervention and control groups. **Conclusions**: Physiotherapy may represent a beneficial complementary approach in migraine management, particularly for improving pain intensity and disability outcomes. However, the available evidence is limited by significant heterogeneity in intervention protocols, outcome measures, and follow-up duration, as well as the lack of quantitative synthesis. Further high-quality randomized trials with standardized methodology and longer follow-up periods are required to strengthen the evidence base.

## 1. Introduction

Migraine is defined as a primary headache disorder [1]. Among all neurological disorders, it is recognised as the leading cause of disability in individuals under 50 years of age [2]. It is characterised by a significant health burden and a reduction in quality of life. Affecting approximately 14.4% of the global population [3], it poses a serious public health challenge in many countries.

Migraine remains an insufficiently understood condition; consequently, patients often encounter inadequate diagnostic and therapeutic procedures [4]. It is estimated that in approximately 25% of patients, migraine attacks are accompanied by ‘aura’-transient neurological symptoms, such as visual, sensory, speech, or motor disturbances [4]. Migraine can present with varying degrees of severity. Approximately 2.5% of patients suffer from chronic migraine [5], defined as the occurrence of headache on more than 15 days per month [1]. Furthermore, migraine is frequently diagnosed as a comorbidity alongside other conditions which, combined with epidemiological data, justifies the need for individualised and multidisciplinary therapeutic approaches.

Standard migraine management relies primarily on pharmacotherapy; however, this often leads to medication-related side effects and, crucially, may not offer sustainable long-term solutions for patients [6,7]. The challenge of treating migraine—like the condition itself—is multifaceted. Consequently, there is growing interest in alternative, non-pharmacological management strategies, particularly physiotherapy. Physiotherapeutic interventions, such as exercise therapy and manual therapy, are frequently employed to reduce co-existing or secondary musculoskeletal dysfunctions that may exacerbate the primary symptom of migraine—pain [8]. Although the number of randomised clinical trials (RCTs) in this field is increasing, there is a lack of evidence synthesis evaluating and comparing the effectiveness of different physiotherapeutic approaches in the treatment of migraine.

Migraine pathophysiology is complex and involves activation of the trigeminovascular system, central sensitization processes, and altered pain modulation within the central nervous system. These mechanisms contribute to both peripheral and central amplification of nociceptive signalling, which may explain the chronic and recurrent nature of migraine attacks.

Previous systematic reviews have predominantly synthesized physiotherapeutic interventions across broad, heterogeneous cohorts, frequently conflating migraine with tension-type or cervicogenic headaches. More recent reviews have either narrowed their scope exclusively to chronic migraine populations (such as Onan et al. [9]) or restricted their analyses purely to exercise modalities via network meta-analysis (such as Reina-Varona et al. [10]). In contrast, the present systematic review evaluates clinical trials across the full spectrum of migraine classifications, synthesizing both active exercise regimens and passive manual therapy modalities published between 2015 and 2025 to clarify their distinct clinical utility.

## 2. Materials and Methods

### 2.1. Protocol and Reporting Standards

This systematic review was conducted and reported in accordance with the Preferred Reporting Items for Systematic Reviews and Meta-Analyses (PRISMA) 2020 guidelines [11]. Although the review protocol was not prospectively registered due to time constraints and the exploratory nature of the clinical question, all methodological stages—including eligibility criteria, search strategy, and outcome selection—were predefined prior to the study’s commencement and remained unchanged throughout the review process. This approach was intended to ensure transparency and minimize the risk of reporting bias.

### 2.2. Eligibility Criteria

Eligibility criteria were defined using the PICOS framework.

1. Population (P): Adults diagnosed with migraine according to the International Classification of Headache Disorders (ICHD-3, ICHD-3 beta, or ICHD-2, depending on the trial’s publication year).
2. Intervention (I): Physiotherapy interventions including manual therapy, exercise therapy or other physiotherapeutic approaches used as standalone or complementary treatments.
3. Comparison (C): Standard pharmacological care, placebo, sham therapy or no intervention.
4. Outcomes (O): Clinical outcomes related to migraine, including attack frequency, pain intensity, attack duration and migraine-related disability (e.g., MIDAS, HIT-6).
5. Study design (S): Randomized controlled trials.

For trials published between 2015 and 2018, prior to the official release of the ICHD-3 guidelines, diagnoses based on the preceding ICHD-2 or ICHD-3 beta criteria were considered eligible.

Studies involving other primary headache disorders (e.g., tension-type headache), observational studies, systematic reviews and meta-analyses were excluded.

### 2.3. Search Strategy

A systematic search of the literature was performed in the following electronic databases: PubMed, Cochrane Library, Scopus and PEDro. The search covered studies published between January 2015 and March 2025.

The search strategy combined terms related to migraine, physiotherapy and randomized controlled trials using Boolean operators. The detailed search strategy, including the specific concepts and keywords used (with an example for the PubMed database), is presented in Table 1.

Reference lists of included studies were also screened to identify additional eligible publications.

### 2.4. Study Selection

All identified records were imported into a database and duplicates were removed. Titles and abstracts were screened independently by two reviewers. Full texts of potentially eligible studies were subsequently assessed according to predefined inclusion and exclusion criteria. Any disagreements between reviewers were resolved through discussion. No artificial intelligence tools or automated machine-learning algorithms were utilized for study identification, record screening, data extraction, or quality appraisal.

### 2.5. Data Extraction

Data from included studies were extracted using a standardized data extraction form. Extracted variables included: author, year of publication, sample size, characteristics of participants, intervention protocol, comparison group, outcome measures, main findings and follow-up duration.

### 2.6. Methodological Quality Assessment

Methodological quality of the included randomized controlled trials was assessed using the Physiotherapy Evidence Database (PEDro) scale. The PEDro scale evaluates criteria such as random allocation, concealed allocation, blinding, and adequacy of follow-up. Scores range from 0 to 10, with higher scores indicating better methodological quality.

### 2.7. Data Synthesis

Due to heterogeneity in intervention protocols, outcome measures and follow-up duration, a quantitative meta-analysis was not conducted. Therefore, results were synthesized narratively, focusing on the direction and magnitude of clinical outcomes across the included studies. A formal meta-analysis was not conducted due to significant methodological and clinical heterogeneity among the included studies. The differences concerned the types of interventions (manual therapy, exercises, mixed approaches), treatment duration, control groups, and various outcome measures used (e.g., MIDAS, HIT-6, attack frequency, pain intensity). Furthermore, variations in follow-up periods and statistical reporting formats precluded a reliable quantitative synthesis of the data. In addition to the narrative synthesis, a semi-quantitative approach based on the direction of effect was employed. Studies were categorized according to whether they demonstrated statistically significant improvement, no difference, or inconclusive results regarding clinical endpoints. This approach enabled a structured comparison of outcomes despite the significant heterogeneity of the study designs.

### 2.8. Use of Artificial Intelligence

During the preparation of this manuscript, the authors utilized the Gemini 1.5 Pro large language model (Google LLC, Mountain View, CA, USA) strictly for the purpose of language polishing, stylistic refinement, and ensuring the accuracy of British Medical English terminology. No artificial intelligence tools were employed in conducting the systematic search, study selection, data extraction, methodological quality assessment, or data synthesis. The authors carefully reviewed all AI-assisted language corrections and take full responsibility for the final content and scientific integrity of this publication.

## 3. Results

### 3.1. Study Selection Process

In the initial phase, 35 duplicates were removed, yielding 249 unique records eligible for title and abstract screening. Subsequently, 235 articles were excluded due to failure to meet the inclusion criteria (PICOS). The main reasons for exclusion were:

(1) The studies were not randomised controlled trials (RCTs).
(2) The target population concerned other headache disorders (e.g., TTH) rather than migraine.
(3) The experiment did not include a comparison with a designed control group (placebo, standard pharmacological care, or a standard physiotherapeutic approach used in both the control and study groups).

**Figure 1:**
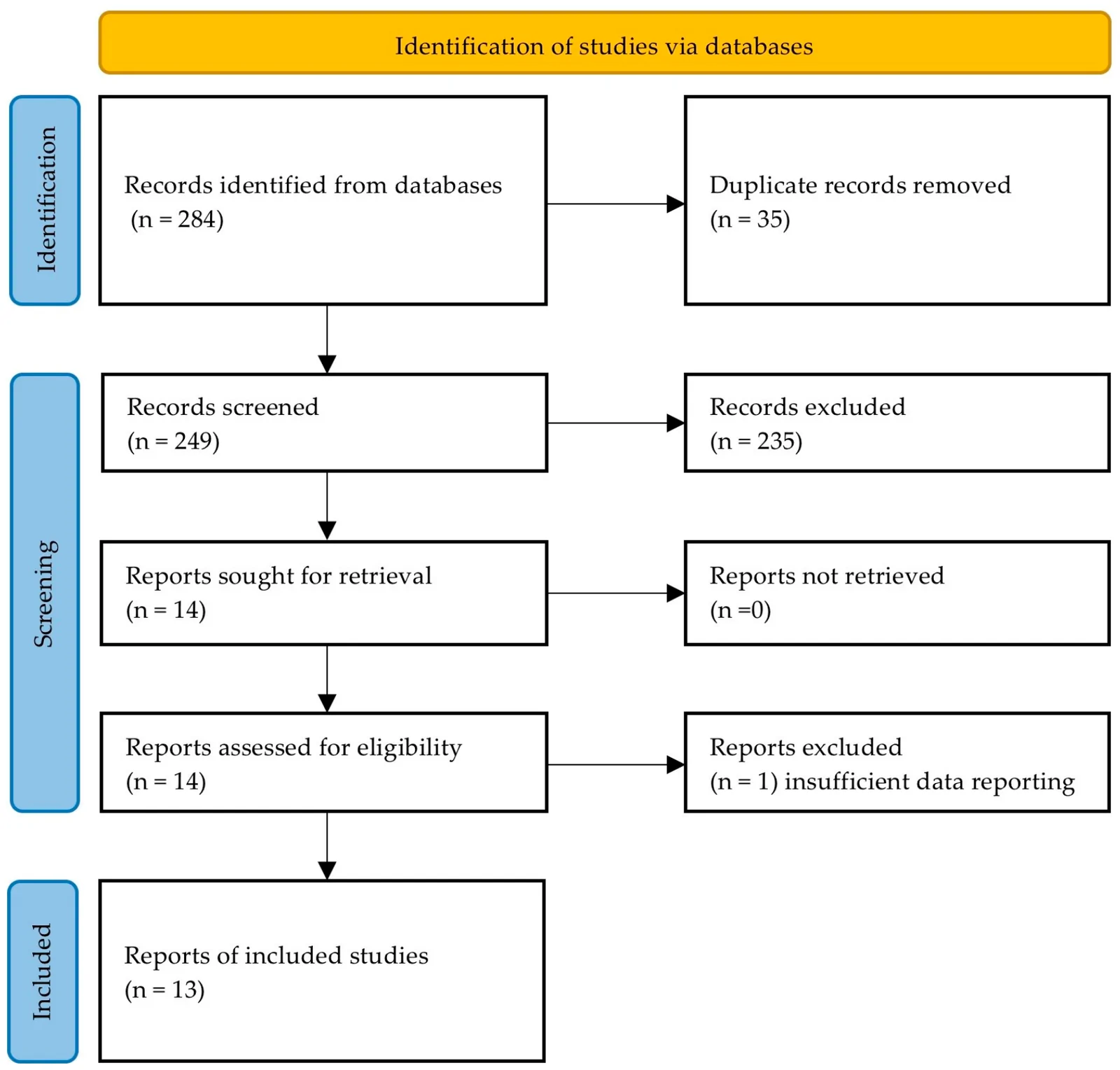
PRISMA flow diagram of the study selection process.

Consequently, 14 full-text articles were assessed for eligibility. One study was excluded due to insufficient data reporting. Ultimately, thirteen (*n* = 13) clinical trials were included in the qualitative analysis, consisting of twelve randomised controlled trials (RCTs) and one preference-based trial [12,13,14,15,16,17,18,19,20,21,22,23,24] (Figure 1).

### 3.2. Study Characteristics

A total of 1021 patients with diagnosed migraine participated in the 13 analysed studies. The mean sample size across the studies was 79 patients. The most frequently applied interventions were manual therapy and exercise therapy. Detailed characteristics of the included studies, including intervention protocols and outcome measures, are summarized in Table 2.

### 3.3. Methodological Quality

The included studies were characterised by satisfactory methodological quality. The mean score on the PEDro scale [25] for the included studies was ≈6.4/10, indicating acceptable methodological rigour. The most frequently identified flaw was the lack of participant blinding, which is standard for physiotherapeutic interventions. Individual scores for each PEDro criterion are presented in Table 3, while the summarized risk of bias assessment for the included trials is illustrated in Table 4.

### 3.4. Clinical Outcomes

A statistically significant and clinically meaningful improvement in at least one of the primary outcomes (frequency, intensity, MIDAS/HIT-6) was observed in 8 studies in the physiotherapy intervention groups compared to the control groups. Reduction in migraine attack frequency was a key positive outcome, recorded in 4 out of 13 studies. Furthermore, migraine attack frequency was the most common primary outcome among the analysed studies.

Beyond statistical significance, several studies demonstrated clinically relevant improvements, particularly in pain intensity and disability scores. Although effect sizes were not consistently reported across all trials, the magnitude of improvement observed in manual therapy interventions suggests at least moderate clinical importance in selected outcomes.

## 4. Discussion

### 4.1. Synthesis of Scientific Evidence

The present systematic review suggests that physiotherapy may be an effective method in migraine management. Analysis of thirteen clinical trials (including 12 RCTs) of satisfactory methodological quality showed that interventions targeting musculoskeletal dysfunctions lead to clinically significant improvement in pain intensity and/or a reduction in the level of functional disability in patients. The best-documented effectiveness was demonstrated for manual therapy interventions which, compared to control/placebo, also contributed to a significant reduction in attack frequency and acute medication intake. These findings align with the meta-analysis by Maistrello et al. who confirmed the effectiveness of manual treatment targeting trigger points in primary headaches [26]. Additionally, Moore et al. highlighted that patients report high satisfaction and perceived effectiveness with manual therapies, which supports the clinical relevance of our results [27].

#### Inconsistency in Intervention Efficacy

It is important to note that not all physiotherapeutic interventions demonstrated consistent effectiveness. Several studies found no statistically significant differences between intervention and control groups, particularly in protocols based primarily on physical exercise. This inconsistency may indicate that the efficacy of physiotherapy depended on the specific type of intervention, patient characteristics, or study design. Furthermore, non-specific effects, including the placebo response associated with manual contact, cannot be entirely ruled out.

### 4.2. Frequency vs. Intensity

The analysis showed that physiotherapy effects were varied, raising the question of which clinical parameter—frequency or intensity—should take precedence in future RCTs. Although pain intensity (measured by the VAS scale) is more subjective, attack frequency is strongly correlated with the diagnosis and is a key indicator of effectiveness in chronic diseases. According to ICHD criteria, the reduction in migraine days should be the main, standardised primary outcome, enabling an objective assessment of intervention effectiveness. Furthermore, it is necessary to unify the observation period in which this frequency is measured. For this purpose, future studies should consider a standard migraine diary, which would serve as a source of information primarily on the frequency, but also the intensity of attacks. Future randomized controlled trials should prioritize migraine frequency (measured as the number of migraine days per month) as the primary endpoint with standardized follow-up periods. Secondary endpoints should include validated disability scales, such as MIDAS or HIT-6, to ensure comparability across studies.

### 4.3. Preventive Action

The unique prophylactic aspect of physiotherapeutic methods (e.g., exercise therapy, relaxation, breathing training) should be emphasised. These interventions, unlike pharmacological drugs, have a positive impact not only on migraine symptoms themselves but also on the general condition of the body, physical capacity, and mental well-being. It is also worth noting that beyond manual interventions, general aerobic exercise has been proven to significantly reduce the number of migraine days, as demonstrated by Lemmens et al. [28]. This suggests that the mechanism of improvement may involve general pain modulation mechanisms, not just biomechanical correction. Long-term RCTs should consider this multimodal aspect of benefits for the patient, as it constitutes significant added value and is key to integrating physiotherapy as a permanent element of the therapeutic procedure for the migraine patient.

### 4.4. Limitations

Several limitations should be considered when interpreting the findings of this review. First, the included randomized controlled trials generally involved relatively small sample sizes, often fewer than 100 participants, which increases the risk of random error and may lead to overestimation of treatment effects. Second, substantial heterogeneity was observed across studies with respect to intervention protocols, including manual therapy, exercise therapy, and multimodal physiotherapeutic approaches that differed in intensity, duration, and therapeutic components. Consequently, the term “physiotherapy” in this context represents a broad group of interventions rather than a single standardized treatment modality. Furthermore, outcome measures varied considerably between studies, with different trials assessing migraine frequency, pain intensity, disability indices, or quality-of-life measures. This variability limits direct comparability and restricts the possibility of quantitative synthesis. Finally, several studies reported neutral or non-significant findings, which should be interpreted alongside positive results to provide a balanced assessment of the current evidence base. Taken together, these factors highlight the need for larger, well-designed randomized trials with standardized outcome measures and longer follow-up periods.

Another limitation is the lack of prospective protocol registration, which may increase the risk of selective reporting bias. Although the methodology was predefined and strictly followed, prospective registration would have further strengthened the transparency and reproducibility of the review process.

A notable methodological and clinical consideration in this review is the inclusion of the trial by Sun et al. (2022) [24], which investigated resistance exercise in vestibular migraine (VM) and contributed substantially to the total synthesized sample size (*N* = 312). Vestibular migraine occupies a unique position in headache classification: while formally recognized in the Appendix of the ICHD-3 and defined through joint consensus criteria by the Bárány Society and the International Headache Society (Lempert et al. [29]), it presents a distinct pathophysiological and clinical phenotype compared to classic migraine. Clinically, VM attacks are characterized predominantly by recurrent episodes of vestibular vertigo, motion sensitivity, and postural imbalance, rather than cephalic pain alone. Consequently, trial endpoints for VM prioritize specialized vestibular instruments—such as the Dizziness Handicap Inventory (DHI) and the Vertigo Severity Scale (VSS)—which are methodologically non-comparable to classic cephalic migraine parameters (e.g., monthly migraine days, MIDAS, HIT-6).

From an epidemiological and therapeutic perspective, recent clinical reviews emphasize that physical therapy for VM specifically targets central vestibular compensation, habituation, and sensory re-weighting, representing a distinct therapeutic mechanism from the musculoskeletal, biomechanical, and trigeminocervical pathways targeted by manual therapy and neck exercises in classic migraine.

From a qualitative sensitivity perspective, when the Sun et al. [24] cohort is appraised independently, the remaining 12 clinical trials (*N* = 709) exclusively represent primary cephalic migraine cohorts. Because the present review performed a narrative synthesis rather than a pooled statistical meta-analysis, the large sample size of Sun et al. did not mathematically weight, skew, or distort the summary effects of the other trials. Importantly, the core qualitative findings across the 12 primary migraine trials remain entirely robust: targeted manual therapy and neck exercises consistently demonstrate clinically meaningful reductions in headache frequency, pain intensity, and migraine-related disability, whereas isolated general exercise yields more variable clinical outcomes across cohorts. Nonetheless, the conceptual divergence of VM highlights the need for future clinical trials and systematic syntheses to evaluate vestibular migraine as a distinct subcategory with dedicated diagnostic and functional criteria.

An additional limitation is the restricted scope of the statistical analysis. Due to the heterogeneity of study designs and outcomes, a formal quantitative synthesis was not possible. Consequently, the analysis was limited to a narrative approach without calculating pooled effect sizes, confidence intervals, or measures of inter-study variability. Moreover, some trials provided insufficient statistical data (e.g., lack of standard deviations or exact effect estimates), which prevented secondary calculations. As a result, the strength and precision of the estimated treatment effects could not be quantitatively assessed, highlighting the need for more consistent reporting in future research.

#### 4.4.1. Standardisation of Assessment Tools

A key challenge in generalising clinical recommendations, including physiotherapeutic ones, is methodological heterogeneity of studies, which is a consequence of the lack of standardised procedures for assessment and work with the migraine patient. The diversity of assessment tools observed in Table 2 (including MIDAS, HIT-6, VAS, SF-36) [12,18,20], and consequently of interventions (manual therapy, exercise therapy, relaxation techniques) [12,18,20], makes meta-analysis impossible and thus reduces the strength of evidence. The lack of a consistent patient assessment standard significantly hinders reliable comparison of different therapeutic methods. This constitutes a serious systemic gap, because if there is no standard of assessment, there cannot be a standard of practice. A presumed standard of assessment should extend beyond simple pain questionnaires and include both symptom diaries [14,19] (which should also be subject to standardisation) and objective biomechanical assessment [13,23] (e.g., assessment of muscle groups in the head and neck area, basic mobility tests, pain assessment) as well as unified interview questions. If the research protocol required a standardised template, researchers could more accurately compare the effectiveness of presented interventions.

#### 4.4.2. Correlation of Adopted Parameters

Effective interpretation of results in physiotherapy requires a clear link between diagnostic and therapeutic elements. If a researcher applies an intervention (e.g., stretching) [13,22] to treat migraine symptoms, the protocol must include an objective measurement of change (e.g., muscle length) both before and after the intervention. Simultaneously, these protocols should focus on diagnostic parameters specific to migraine (e.g., MIDAS), treating, for example, muscle shortening as a secondary condition, the overcoming of which may prove beneficial for recovery, but will not constitute evidence of improvement in the migraine itself [13,22]. Such a methodology is essential to establish what actually influenced the clinical result and to avoid a situation where a positive result is attributed to an intervention whose mechanism of action was not proven within the same study. In the future, RCTs must more clearly demonstrate the cause-and-effect relationship between the deficit and the intervention undertaken to increase the credibility of protocols.

#### 4.4.3. Risk of Bias Correlated with the Comparison Group

From the perspective of methodological rigour, the best approach to this type of experiment is to compare physiotherapeutic interventions with a reliable placebo (sham treatment) or standard pharmacotherapy. Some of the included RCTs examined active physiotherapy methods. It should be noted that comparing several techniques requires the researcher to isolate the research environment and create groups that will correlate with each other in an appropriate way, e.g., chiropractic therapy + standard care vs. chiropractic therapy. Unfortunately, comparing several methods in one study carries the risk of distorting results. Furthermore, as noted by Rist et al., the quality of evidence for spinal manipulation is often moderate, and the non-specific effects (placebo) of manual contact are difficult to entirely rule out in such trials [30]. Achieving a statistically significant difference between two active forms of treatment is more difficult, which may lead to false conclusions regarding a lack of effectiveness or, conversely, overestimated effectiveness of a given method, while in reality, both methods may be equally effective or ineffective compared to a control group. Therefore, in the record identification process, studies comparing different active methods without distinguishing a group that would constitute a control group and ensure a reliable reference point were consciously excluded. To assess the actual effectiveness of physiotherapy methods, future studies should aim for Intervention vs. Placebo protocols, thus minimising the influence of confounding factors.

#### 4.4.4. Standardisation of the Follow-Up Phase

Another, equally serious methodological limitation is the lack of standardisation regarding the length of the follow-up period. As the analysis shows (Table 2), most included studies incorporated long-term effect measurement in their protocol [12,20]; however, their observation period was relatively short [21,22] or differed significantly in length between individual studies. A total of 7 out of 13 studies included a follow-up longer than 3 months. Considering the chronic nature of migraine and the fact that a feature of physiotherapeutic interventions is long-term action (and clinical improvement often does not occur immediately after the intervention ends), the lack of follow-ups or a short monitoring period is tantamount to wasting or underutilising the full research potential of the experiment. From a practical point of view, the assessment of intervention effectiveness in chronic diseases, such as migraine, must be based on evidence that effects are sustained for at least 3 to 6 months [12,20]. The necessity of introducing mandatory long-term follow-up (2–3 follow-up visits at 3, 6, and 12 months after study participation ends) should be a key recommendation for future RCTs in the field of physiotherapy for migraine patients.

### 4.5. Strengths of the Review

This systematic review has several strengths that enhance the reliability of its findings. It was conducted in accordance with the PRISMA 2020 guidelines, ensuring a transparent and reproducible selection process. The completed PRISMA 2020 checklist and Search Strategy are provided in Supplementary File S1. A comprehensive search strategy across multiple databases (PubMed, Cochrane Library, Scopus, and PEDro) minimized the risk of omitting relevant studies. Furthermore, the use of strict PICOS eligibility criteria and the methodological assessment via the PEDro scale provided an objective evaluation of the evidence base and risk of bias.

### 4.6. Quality of Evidence

Although a formal GRADE assessment was not performed, the overall quality of evidence can be considered low to moderate. This is primarily due to methodological limitations in the included trials, such as small sample sizes, lack of blinding (particularly for participants and therapists), and variability in intervention protocols. The heterogeneity of outcome measures and follow-up periods further limits the consistency of the results. Consequently, while the evidence suggests a beneficial effect of physiotherapy in migraine, the certainty of these findings remains limited.

## 5. Conclusions

The findings of this systematic review suggest that physiotherapy may represent a beneficial complementary approach in the management of migraine. Several randomized controlled trials reported improvements in migraine-related outcomes, including reductions in pain intensity, attack frequency and disability scores. However, the available evidence is characterized by methodological heterogeneity in intervention protocols, outcome measures and follow-up duration.

Therefore, although physiotherapy shows promising clinical potential, further high-quality randomized controlled trials with standardized protocols and longer follow-up periods are necessary to strengthen the current evidence base.

## Figures and Tables

**Table 1:** Search Strategy.

Concept	Search Terms (Keywords & Synonyms)	Boolean Operator
Condition	migraine OR “migraine disorder” OR “migraine headache”	AND
Intervention	physiotherapy OR “physical therapy” OR “rehabilitation” OR “exercise therapy” OR “manual therapy”	AND
Study Design	“randomized controlled trial” OR randomized OR RCT	-

**Table 2:** Characteristics of included studies.

Author (Year)	Participants (N)	Intervention (I) vs. Control (C)	Outcome Measures	Main Results (O)	Follow-Up
Krøll (2018) [12]	52	Exercise Therapy (ET) vs. No intervention	Number of migraine symptom days	No statistically significant differences between groups.	At 6 months - No statistically significant differences between groups.
Rezaeian (2021) [13]	40	Manual Therapy (MT) vs. Placebo	Pain intensity, Neck Disability Index (NDI); Cervical Range of Motion (CROM)	Statistically significant difference between groups in outcome improvement across all three parameters, favouring MT (*p* < 0.001).	At 1 month - Statistically significant difference between groups in outcome improvement across all three parameters, favouring MT (*p* < 0.001).
Bevilaqua-Grossi (2016) [14]	50	Standard pharmacological care (SPC) + Manual therapy (MT) vs. Standard pharmacological care (SPC)	Headache frequency	- No statistically significant differences between groups. - Improvement observed in both groups.	At 1 month - No statistically significant differences between groups.
Meise (2023) [15]	82	Standard of physiotherapy (SoP) + Pain neuroscience education (PNE) vs. Standard of physiotherapy (SoP)	Headache frequency; migraine frequency; Migraine Associated Disability Score (MIDAS)	- Reduction in headache frequency in both groups. No statistically significant differences between groups. - Statistically significant difference in migraine frequency between groups (*p* = 0.008), favouring PT + PNE. - Reduction in MIDASs in both groups. No statistically significant differences between groups.	At 3 months - No statistically significant difference between groups in headache frequency. - Statistically significant difference in the reduction in migraine frequency (*p* = 0.003), favouring the PT + PNE group. - No statistically significant differences between groups in MIDASs.
Garrigós-Pedrón (2018) [16]	45	Standard pharmacological care (SPC) + exercise therapy (ET) + Cervical group (CG) vs. Standard pharmacological care (SPC) + exercise therapy (ET) + Cervical and orofacial group (COG)	Craniofacial Pain and Disability Inventory (CF-PDI); Headache Impact Test (HIT-6)	- Statistically significant reduction in CF-PDI in both groups. Statistically significant difference between groups (*p* = 0.027). - Statistically significant reduction in HIT-6 in both groups. Statistically significant difference between groups (*p* < 0.001).	At 12 weeks - Statistically significant difference in CF-PDI reduction between groups (*p* = 0.042), favouring COG. - Statistically significant difference in HIT-6 reduction between groups (*p* = 0.002), favouring COG.
Muñoz-Gómez (2021) [17]	50	Manual Therapy (MT) vs. Placebo	Pain intensity; MIDAS; Physical Quality of Life (PQoL); Overall Quality of Life (OQoL); reduction in medication intake; The Patients’ Global Impression of Change (PGIC)	- Statistically significant difference between groups in pain intensity reduction, favouring MT (*p* < 0.001). - Statistically significant difference between groups in MIDAS reduction, favouring MT (*p* < 0.001). - Statistically significant difference between groups in the improvement of PQoL and OQoL outcomes (*p* < 0.05). - Statistically significant difference between groups in the reduction in medication intake, favouring MT (*p* < 0.001). - No statistically significant differences in PGIC in either group.	At 1 month - Statistically significant difference between groups in pain intensity reduction, favouring MT (*p* < 0.001). - Statistically significant difference between groups in MIDAS reduction, favouring MT; medium effect size. - Statistically significant difference between groups in the improvement of PQoL and OQoL outcomes (*p* < 0.001); medium effect size. - Statistically significant difference between groups in the reduction in medication intake, favouring MT (*p* < 0.05); medium effect size. - No statistically significant differences in PGIC in either group.
Muñoz-Gómez (2022) [18]	50	Manual Therapy (MT) vs. Placebo	Pain intensity; migraine frequency and severity (Headache Disability Index (HDI)); functional, emotional, and overall disability (HDI); medication intake; PGIC	- Statistically significant difference between groups in pain intensity reduction, favouring MT (*p* < 0.05). - No statistically significant differences between groups in migraine severity. - Statistically significant difference between groups in migraine frequency reduction, favouring MT (*p* < 0.05). - Statistically significant difference between groups in the reduction in overall and functional HDI, favouring MT (*p* < 0.05). - No statistically significant differences between groups in emotional HDI reduction. - Statistically significant difference between groups in medication intake reduction, favouring MT (*p* < 0.05). - Statistically significant difference between groups in PGIC improvement favouring MT (*p* < 0.01).	At 8 weeks - Statistically significant difference between groups in pain intensity reduction, favouring MT (*p* < 0.05). - No statistically significant differences between groups in migraine severity. - Statistically significant difference between groups in migraine frequency reduction, favouring MT (*p* < 0.05). - Statistically significant difference between groups in the reduction in overall and functional HDI, favouring MT (*p* < 0.05). - No statistically significant differences between groups in emotional HDI reduction. - Statistically significant difference between groups in medication intake reduction, favouring MT (*p* < 0.05). - Statistically significant difference between groups in PGIC improvement, favouring MT (*p* < 0.001).
Luedtke (2020) [19]	103	Manual Therapy (MT) vs. Exercise therapy (ET) (as SoP)	Headache frequency during the 4 weeks post-intervention	No statistically significant differences between groups.	At 3 months - No statistically significant differences between groups.
Chaibi (2016) [20]	104	Manual Therapy (MT) vs. Standard pharmacological care (SPC) or Placebo	Number of migraine days per month	- Statistically significant difference between MT and SPC groups in the reduction in migraine days, favouring MT (*p* = 0.02). - No statistically significant differences between MT and placebo groups.	At 3, 6 and 12 months - Statistically significant difference between the MT group and SPC group in the reduction in migraine days, favouring the MT group across all time points (*p* < 0.025). - No statistically significant differences between MT and placebo groups.
Rist (2021) [21]	61	Manual therapy (MT) + Enhanced usual care (EUC) vs. Enhanced usual care (EUC)	Change in the number of migraine days	Statistically significant difference between groups in the reduction in migraine days, favouring MT + EUC (*p* < 0.05).	At 4 weeks - Statistically significant difference between groups in the reduction in migraine days, favouring MT + EUC (*p* < 0.05).
Wanderley (2020) [22]	30	Manual Therapy (MT) vs. Exercise therapy (ET) (as SoP)	Headache frequency; pain intensity; number of days with maximum pain during each headache attack; duration of a single attack; PGIC	No statistically significant differences between groups.	At 30 days - No statistically significant differences between groups.
Benatto (2022) [23]	42	Exercise Therapy (ET) vs. Placebo	Headache frequency; headache intensity	No statistically significant differences between groups.	At 3 months - No statistically significant differences between groups.
Sun (2022) [24]	312	Exercise Therapy (ET) vs. Placebo (relaxation control group)	Dizziness Handicap Inventory (DHI); Vertigo Severity Scale (VSS); number of vertigo attacks during the last week	No data available from End Of Treatment	At 2 months - Statistically significant difference between groups in DHI reduction, favouring exercise therapy (*p* = 0.02). - No statistically significant differences between groups in VSS or the number of vertigo attacks during the last week. At 4 months - Statistically significant difference between groups in DHI reduction, favouring exercise therapy (*p* = 0.01). - Statistically significant difference between groups in the reduction in the number of vertigo attacks during the last week (*p* = 0.02). - Statistically significant difference between groups in VSS reduction, favouring exercise therapy (*p* = 0.03).

**Table 3:** Detailed methodological quality assessment based on the PEDro scale.

Author (Year)	Randomization	Allocation	Blinding	Follow-Up	Pedro Score
Krøll (2018) [12]	Yes (Computer-generated block randomization)	Concealed (Sealed, consecutively numbered envelopes)	Single-blinded (Assessor blinded)/No patient blinding	Post-treatment and at 1-month follow-up	5
Rezaeian (2021) [13]	Yes (Block randomisation methods)	Not clearly reported	Likely single-blinded (Placebo-controlled)	Post-treatment and at 1-month follow-up	5
Bevilaqua-Grossi (2016) [14]	Yes (Simple 1:1 randomisation sequence)	Concealed (Sealed opaque envelopes prepared by an independent researcher)	Single-blinded (Assessor blinded)/No patient blinding	Post-treatment and at 1-month follow-up	6
Meise (2023) [15]	Yes (Simple 1:1, unrestricted randomisation)	Concealed (Sealed opaque envelopes chosen by participants)	Non-blinded	Post-treatment and at 3-month follow-up	6
Garrigós-Pedrón (2018) [16]	Yes (Computer-generated stratified randomisation)	Concealed (Assigned by an independent study member)	Single-blinded (Outcome assessor blinded)	Post-treatment, at 6 and 12-week follow-up	6
Muñoz-Gómez (2021) [17]	Yes (Simple 1:1 randomisation)	Concealed (Sealed, sequentially numbered envelopes)	Double-blinded (Patients and statistician blinded)	Post-treatment and at 1-month follow-up	7
Muñoz-Gómez (2022) [18]	Yes (Computer-generated block randomisation)	Not clearly reported	Single-blinded (Placebo controlled)	Post-treatment and at 8-week follow-up	6
Luedtke (2020) [19]	No (Preference-based clinical trial)	Not applicable	Non-blinded	Post-treatment and at 3-month follow-up	Not applicable
Chaibi (2016) [20]	Yes (Stratified block randomisation by drawing lots)	Concealed (Numbered sealed lots administered by an external party)	Single-blinded (Patient blinding success assessed via questionnaire)	Post-treatment, at 3, 6 and 12-month follow-up	8
Rist (2021) [21]	Yes (Computer-generated stratified random permuted block randomisation, 1:1 ratio)	Not clearly concealed (Investigator-generated scheme, assigned by assistant)	Non-blinded	Post-treatment	7
Wanderley (2020) [22]	Yes (Simple randomisation using a table of random numbers, 1:1:1 ratio)	Concealed (Sealed opaque envelopes)	Single-blinded (Assessor blinded)	Post-treatment	7
Benatto (2022) [23]	Yes (Simple 1:1 randomisation sequence)	Concealed (Sealed opaque envelopes)	Double-blinded (Outcome assessor and therapist)	Post-treatment and at 3-month follow-up	8
Sun (2022) [24]	Yes (Stratified block permutation)	Concealed (Evaluators unaware of group assignment)	Single-blinded (Outcome assessor blinded)	Post-treatment, at 2 and 4-month follow-up	6

**Table 4:** Summary of the risk of bias assessment for the included studies.

Author (Year)	Main Risk of Bias
Krøll (2018) [12]	High performance bias (lack of patient/therapist blinding) and selection bias (unclear allocation details).
Rezaeian (2021) [13]	Selection bias (allocation concealment not reported) and performance bias.
Bevilaqua-Grossi (2016) [14]	Performance bias (non-blinded subjects) but low detection bias (blinded assessor).
Meise (2023) [15]	High performance bias (open-label design; participants aware of intervention).
Garrigós-Pedrón (2018) [16]	Performance bias (subject blinding not feasible) and limited long-term data.
Muñoz-Gómez (2021) [17]	Performance bias (non-blinded therapist) but low selection and detection bias.
Muñoz-Gómez (2022) [18]	Selection bias (unclear allocation concealment) and lack of subject blinding.
Luedtke (2020) [19]	High selection bias (non-randomised, preference-based design) and performance bias.
Chaibi (2016) [20]	Low risk overall; main risk is unavoidable non-blinded therapist in SMT.
Rist (2021) [21]	Selection bias (allocation not clearly concealed) and high-performance bias (non-blinded).
Wanderley (2020) [22]	Performance bias (single-blinded) and potential therapist bias (only one provider).
Benatto (2022) [23]	Low risk overall; excellent concealment and assessor blinding; unavoidable non-blinded therapist.
Sun (2022) [24]	Performance bias (subjects not blinded) and lack of detailed allocation concealment.

## Data Availability

No new data were created or analyzed in this study. Data sharing is not applicable to this article.

## Supplementary Materials

The following supporting information can be downloaded at: https://www.mdpi.com/article/10.3390/clinpract16100192/s1, File S1: PRISMA 2020 checklist and Search Strategy.

## Abbreviations

The following abbreviations are used in this manuscript:

CF-PDI	Craniofacial Pain and Disability Inventory
CG	Cervical Group
COG	Cervical and Orofacial Group
CROM	Cervical Range of Motion
DHI	Dizziness Handicap Inventory
ET	Exercise Therapy
EUC	Enhanced Usual Care
HDI	Headache Disability Index
HIT-6	Headache Impact Test-6
ICHD	International Classification of Headache Disorders
MIDAS	Migraine Disability Assessment
MT	Manual Therapy
NDI	Neck Disability Index
OQoL	Overall Quality of Life
PEDro	Physiotherapy Evidence Database
PGIC	Patients’ Global Impression of Change
PICOS	Population, Intervention, Comparison, Outcomes, Study design
PNE	Pain Neuroscience Education
PQoL	Physical Quality of Life
PRISMA	Preferred Reporting Items for Systematic Reviews and Meta-Analyses
RCTs	Randomised Controlled Trials
SoP	Standard of Physiotherapy
SPC	Standard Pharmacological Care
TTH	Tension-Type Headache
VAS	Visual Analogue Scale
VSS	Vertigo Severity Scale
SF-36	Short Form-36 Health Survey
