# Supplementary material for: Physiotherapy Interventions for Migraine: A Systematic Review of Clinical Trials
Source: Clin Pract. 2026 Oct 13;16(10):192. doi: 10.3390/clinpract16100192 (PMC13703453; doi:10.3390/clinpract16100192)
Supplement: Supplementary file 1 [file clinpract-16-00192-s001.zip › SEARCH STRATEGY.pdf]

Supplementary File: Detailed Search Strategy

Title: Physiotherapy Interventions for Migraine: A Systematic Review of Clinical Trials

1. General Search Information

The literature search was conducted to identify relevant studies investigating physiotherapeutic interventions for migraine management. The search was performed across four major electronic databases: PubMed, Cochrane Library, Scopus, and PEDro. The search period was defined from January 1, 2015, to March 1, 2025.

2. Detailed Search Strings and Database Results

   a. Database: PubMed

      - Search String: (migraine OR migraine disorder OR migraine headache) AND (physiotherapy OR physical therapy OR rehabilitation OR exercise therapy OR manual therapy) AND (randomized controlled trial OR randomized OR RCT)

      - Filters applied: Publication date: 2015/01/01 to 2025/03/01; Humans; Adults (19+)

      - Results (n): 125

   b. Database: Scopus

      - Search String: TITLE-ABS-KEY ((migraine OR "migraine disorder" OR "migraine headache") AND (physiotherapy OR "physical therapy" OR "rehabilitation" OR "exercise therapy" OR "manual therapy") AND ("randomized controlled trial" OR randomized OR rct))

      - Filters applied: 2015–2025

      - Results (n): 53

   c. Database: Cochrane Library

      - Search String: (migraine OR "migraine disorder"):ti,ab,kw AND (physiotherapy OR "physical therapy" OR exercise OR "manual therapy"):ti,ab,kw

      - Filters applied: 2015–2025; Trials

      - Results (n): 1

   d. Database: PEDro (Physiotherapy Evidence Database)

      - Search String: Abstract & Title: "migraine"

      - Filters applied: Published since 2015; Subdiscipline: neurology; Method: clinical trial

      - Results (n): 105

Total records identified (n): 284

3. Selection Summary

The search strategy focused on identifying clinical trials (primarily RCTs) involving adult populations with migraine. Following the identification of 284 records, 35 duplicates were removed, leaving 249 unique records for title and abstract screening.
